# *Notes from the Field:* Multistate, Multiserotype Outbreak of *Salmonella* Infections Linked to Cashew Brie — United States, 2021

**DOI:** 10.15585/mmwr.mm7221a4

**Published:** 2023-05-26

**Authors:** Kailey Lewis, Michael Vasser, Katie Garman, Jeffrey Higa, Michael Needham, D. J. Irving, Steffany Cavallo, Dominique Sullivan, Marks, Margaret Kirchner, Asma Madad, Zachary D. McCormic, John Dunn

**Affiliations:** ^1^Tennessee Department of Health; ^2^Division of Foodborne, Waterborne, and Environmental Diseases, National Center for Emerging and Zoonotic Infectious Diseases, CDC; ^3^Oak Ridge Institute for Science and Education, Oak Ridge, Tennessee; ^4^California Department of Public Health; ^5^Los Angeles County Department of Public Health, El Monte, California; ^6^Coordinated Outbreak Response and Evaluation Network, Food and Drug Administration, College Park, Maryland.

On March 30, 2021, during weekly analysis of sequenced isolates, the Tennessee Department of Health identified two *Salmonella* Duisburg isolates that had been determined to be closely related by whole genome sequencing (WGS). The specimens containing the isolates were from two patients who reported eating the same brand of cashew brie (a vegan brie cheese alternative) at the same restaurant. A search of the National Center for Biotechnology Information (NCBI) Pathogen Detection Isolates Browser identified three additional *Salmonella* isolates, two from patients in California and one in Florida, that were closely related genetically to the Tennessee isolates. The California Department of Public Health confirmed that one patient consumed the same brand of cashew brie before becoming ill. The Florida Department of Health reported the patient followed a vegan diet, which excluded some potential food exposures. A multistate investigation to characterize illnesses and identify the outbreak source was initiated. Open-source access to WGS data through NCBI facilitated rapid investigation of this outbreak before it was large enough to be identified through standard multistate outbreak detection methods (*1*). Rapid detection, investigation, and product recall prevented additional illnesses.

A case was defined as a *Salmonella* infection with one of four outbreak strains (identified using WGS) with illness onset during December 1, 2020–May 9, 2021 (Figure). State and local officials interviewed patients about the foods they consumed before illness onset, including cashew brie, and where the foods were purchased. Product and environmental sampling were conducted at retail locations or at the sole cashew brie production facility identified during of the Food and Drug Administration (FDA) traceback investigation. Outbreak strains of *S.* Chester, *S*. Typhimurium, and *S*. Urbana were included in the investigation because patients reported consuming cashew brie and cashew brie and component ingredients tested positive for these strains of *Salmonella*. This activity was reviewed by CDC and was conducted consistent with applicable federal law and CDC policy.*

**FIGURE:**
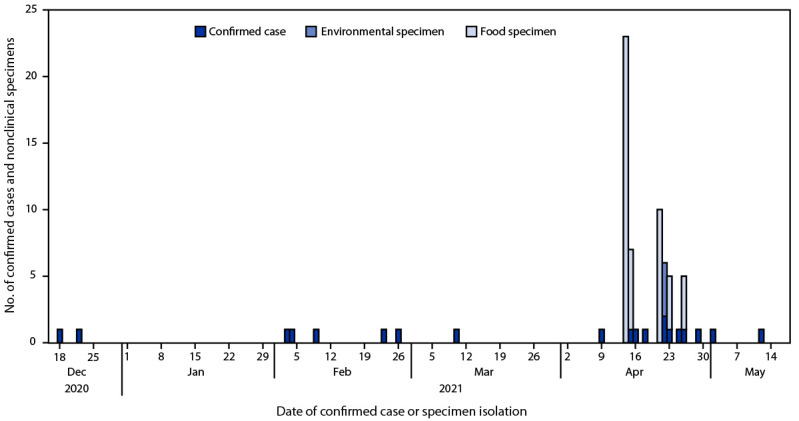
Number of confirmed cases (n = 20) and nonclinical (environmental and food) specimens of *Salmonella* (n = 51) linked to cashew brie by date of isolation — United States, December 2020–May 2021

Overall, 20 cases were identified in four states: California (seven *S*. Typhimurium, three *S*. Chester, three *S*. Urbana, two *S*. Duisburg), Florida (one *S*. Chester, one *S*. Duisburg), Maryland (one *S*. Urbana), and Tennessee (two *S*. Duisburg). The median patient age was 26 years (range = 1–72 years); 65% were female. Five patients were hospitalized, and none died.

Among 19 patients who were interviewed, 15 reported eating the same brand of cashew brie during the week before illness onset. Thirty-six samples were collected by state and federal officials from component ingredients, in-process and finished products, and environmental swabs from the cashew brie production facility. Twenty-three (64%) samples yielded 51 *Salmonella* isolates, including 19 (95%) of 20 retail samples and four (25%) of 16 samples collected from the production facility. On the basis of these findings, the cashew brie producer voluntarily recalled all products. Four *Salmonella* strains were isolated from 51 food and environmental samples; the results of WGS analysis indicated that only *S.* Chester and *S.* Urbana detected in non-clinical samples were associated with human illness. In addition, *S.* Duisburg and *S*. Typhimurium were only isolated from clinical samples and not found in food or environmental samples. 

On the basis of the food sample results and FDA traceback, the cashew ingredients used to make the brie products were the likely source of contamination. Review of cashew brie production revealed no lethality treatment (e.g., pasteurizing or irradiation) (*2*) before cashew processing. FDA worked with the cashew supplier to ensure potentially contaminated cashews were no longer on the market and the supplier implemented corrective actions.

Outbreaks associated with raw nut and seed products are well documented (*3*), and *Salmonella* outbreaks associated with cashew cheese have been reported (*4*). The lack of a lethality treatment for component ingredients can increase the risk of contamination in products that are served ready-to-eat and perceived as safe by the public. The identification of two persons who became ill after eating the same uncommon food at the same restaurant, paired with detection of a rare *S. *Duisburg serotype, led to an early hypothesis about the source of this outbreak. Open-source access to WGS data through NCBI enabled rapid investigation of this outbreak before it was large enough to be identified using the standard multistate outbreak detection methods (*1*). Rapid detection, investigation, and product recall prevented additional illnesses, given the detection of *Salmonella* in 95% of cashew brie products collected at retail locations during this investigation.

## Footnotes

* 45 C.F.R. part 46.102(l)(2), 21 C.F.R. part 56; 42 U.S.C. Sect. 241(d); 5 U.S.C. Sect. 552a; 44 U.S.C. Sect. 3501 et seq.
